# Patient-centered evaluation of an expectation-focused intervention for patients undergoing heart valve surgery: a qualitative study

**DOI:** 10.3389/fcvm.2024.1338964

**Published:** 2024-02-15

**Authors:** Caroline Clifford, Evaldas Girdauskas, Susanne G. R. Klotz, Saskia Kurz, Bernd Löwe, Sebastian Kohlmann

**Affiliations:** ^1^ Department of Psychosomatic Medicine and Psychotherapy, University Medical Center Hamburg-Eppendorf, Hamburg, Germany; ^2^ Department of Cardiothoracic Surgery, University Medical Center Augsburg, Augsburg, Germany; ^3^ Department of Physiotherapy, University Medical Center Hamburg-Eppendorf, Hamburg, Germany

**Keywords:** enhanced recovery after surgery (ERAS), evaluation, expectations, heart valve surgery, preoperative psychological intervention, qualitative research

## Abstract

**Objective:**

Randomized controlled trials demonstrate the effectiveness of expectation-focused interventions in improving recovery outcomes following cardiac surgery. For dissemination in routine health care, it is important to capture the perspective of affected individuals. This qualitative study explores the perceived benefits and intervention-specific needs of patients who received expectation-focused intervention in the context of heart valve surgery. In addition, it explores potential barriers and adverse effects.

**Methods:**

As part of an Enhanced Recovery After Surgery (ERAS) program within a multicentered randomized controlled trial, patients undergoing minimally invasive heart valve surgery received an intervention focused on their expectations. Six weeks after the intervention, semi-structured interviews were conducted with 18 patients to assess its feasibility, acceptance, barriers, benefits, and side effects. The transcribed interviews were analyzed using qualitative content analysis.

**Results:**

The results indicate that both the intervention and the role of the patient and psychologist are key aspects in evaluating the expectation-focused intervention. Five key themes emerged from the patients’ perspective: personal needs, expectations and emotions, relationship, communication, and individuality. Patients valued the preparation for surgery and recovery and the space for emotions. Establishing a trustful relationship and addressing stigmatization were identified as primary challenges within the intervention.

**Conclusion:**

Overall, patients experienced the expectation-focused intervention as helpful and no adverse effects were reported. Perceived benefits included enhanced personal control throughout the surgery and recovery, while the potential barrier of stigmatization towards a psychologist may complicate establishing a trustful relationship. Addressing personal needs, as a relevant topic to the patients, could be achieved through additional research to identify the specific needs of different patient subgroups. Enhancing the expectation-focused intervention could involve the implementation of a modular concept to address individual needs better.

## 1 Introduction

Numerous research findings indicate that patient expectations significantly influence treatment outcomes, irrespective of factors such as demographic variables, experienced stress, socioeconomic status, and health behavior (1, 2). This influence remains consistent regardless of the patient's medical condition or the type of surgical procedure (3). Negative expectations are associated with more complications, lower quality of life, higher illness-related disability, depressive symptoms, and prolonged inability to work (4, 5). Interventions to optimize patients' expectations before undergoing heart surgery have been developed in the past (5–7). Former studies showed an association between expectation-focused interventions and a faster recovery, including a reduced hospital stay and quicker return to work (8–10). Patients reported less postoperative pain and showed better physical health outcomes (10, 11). Furthermore, expectation-focused interventions were associated with greater personal control beliefs, increased quality of life, and reduced cardiac anxiety (12–14).

As research showed the advantages of expectation-focused interventions, Rief and colleagues developed a standardized preoperative intervention known as EXPECT within the PSY-HEART trial. The EXPECT intervention aims at optimizing the patients' expectations in the context of a coronary artery bypass graft surgery (6, 7, 13). This expectation-focused intervention was based on the integrative model of expectations which includes generalized self-efficacy, treatment outcome expectations (benefit expectations), timeline expectations, and personalized outcome expectancy (3).

To further illustrate the effectiveness of expectation-focused interventions in different medical contexts and as the need for psychological support for patients undergoing heart valve surgery was identified in former studies, an expectation-focused intervention based on the EXPECT intervention and adapted for patients with heart valve surgery was conducted (6, 7, 15). The intervention was implemented into an interprofessional Enhanced Recovery After Surgery (ERAS) program within a randomized controlled trial (RCT) on the improvement of treatment for patients undergoing heart valve surgery (16, for the study protocol; main manuscript under preparation). As the ERAS program was part of an RCT, a comparison between patients receiving the expectation-focused intervention and those receiving treatment as usual was possible. The expectation-focused intervention involved sessions led by a psychologist and included the development of realistic expectations about the benefits of the surgery and the recovery process. Relevant steps to achieve the personal goals as well as strategies to handle unpleasant symptoms were discussed (13). The first sessions took place four to six weeks before the surgery, followed by sessions one day before the surgery and during the hospital stay after the surgery. Relevant information and worksheets in a diary designed interprofessionally complemented the intervention and a last follow-up via telephone was conducted six weeks after the operation.

To date, the evaluation of expectation-focused interventions has primarily focused on quantifiable measures such as the duration of hospital stays or patient-reported outcomes such as anxiety (8, 13). To effectively disseminate an intervention, it is crucial to gain insights into the personal needs of patients (17–19). By including patients, we can identify which resources were experienced as needed, which needs were assessed as unmet and we can identify personal needs on a subgroup level (20).

In this study, we aimed to capture the evaluation of patients regarding an expectation-focused intervention in the context of heart valve surgery. Specifically, through a qualitative interview study, we intended to explore which aspects of the expectation-focused intervention are perceived as beneficial by patients undergoing heart valve surgery, whether they encounter any adverse effects, and how the intervention could be further enhanced.

## 2 Material and methods

### 2.1 Study context

The study was conducted as a qualitative follow-up of a randomized controlled trial (RCT) on the improvement of treatment for patients undergoing heart valve surgery (16, for the study protocol; main manuscript under preparation). In this trial, *N* = 186 patients undergoing minimally invasive heart valve surgery were randomized to treatment as usual or to an interprofessional treatment following an Enhanced Recovery After Surgery (ERAS) protocol. Part of the ERAS protocol for the intervention group was an expectation-focused intervention aiming at the development of realistic expectations concerning the surgery and its outcome, preparing for side effects (e.g., pain), and addressing emotions (e.g., anxiety). The medical clarification and decision-making processes such as heart valve choice occurred with the cardiac surgeon and were not part of the expectation-focused intervention. The Department of Psychosomatic Medicine and Psychotherapy conducted the expectation-focused intervention. An interprofessional approach involving surgical, anesthesiological, physiotherapeutic, and advanced nursing components was also part of the ERAS program.

### 2.2 Sampling

Recruitment for the qualitative study took place in Hamburg (Germany) between November 2021 and July 2022. Patients were recruited through a randomized controlled trial on the improvement of treatment for patients undergoing heart valve surgery at the University Medical Center Hamburg-Eppendorf. Inclusion criteria were an indication for elective minimally invasive aortic or mitral valve surgery and a functional status classification as “FIT” or “Pre-Frail” with the LUCAS functional index derived from the Longitudinal Urban Cohort Ageing Study (21). Furthermore, written informed consent, sufficient German language skills, and the ability to adequately understand the nature and extent of the individual's requirement for participation in the ERAS model of care were required. Exclusion criteria were severe chronic obstructive pulmonary disease, dialysis-dependent renal failure, advanced liver cirrhosis, residual neurological impairment following a prior stroke, predicted life expectancy of less than one year, previous cardiac surgery, severe depressive disorder, substance-related addictive disorders or a lack of a social environment that ensures adequate supportive care during the perioperative time.

As a second step, participants received information about the qualitative study before participation and were free to decide whether they wished to participate independently of their participation within the randomized controlled trial on the improvement of treatment for patients undergoing heart valve surgery. All participants gave written informed consent regarding the qualitative study. In addition, patients received an expense allowance of 15 € for participating in the interviews. The qualitative study was conducted in accordance with the Helsinki Declaration and was approved by the Ethics Committee of the University Medical Center (reference number: LPEK-0358).

We applied a purposive sampling strategy to ensure that variations in gender, age, and disease duration were accounted for. We included patients of both, the intervention and control groups, to compare how patients received the expectation-focused intervention and how patients would evaluate their motivation in participating. Considering a high variation of age, gender, and disease duration, we collected a sample size of *N* = 18 patients, based on findings on saturation of themes (22).

### 2.3 Study design and data collection

We used an exploratory, qualitative framework, applying a semi-structured interview guide. The semi-structured interviews with *N* = 18 patients were conducted by the first author (CC). Interviews were split into two parts in order not to overstrain the patients, each lasting between 35 and 45 min. The interviews took place after rehabilitation, about six weeks after the operation, and were conducted virtually via video-telephony as patients lived at various locations across Northern Germany. The semi-structured interview guide included topics addressed in the expectation-focused intervention, such as feasibility, acceptance, barriers, and effectiveness plus the experience of adverse effects. Further key issues regarding the combination of psychology and cardiology in treatment and the perception of the interprofessional approach were also included in the interview guide. The different topics were supplemented by more structured questions based on pre-identified themes. Prompting questions were used to encourage patients to elaborate on their experiences and reduce possible bias by expressing both positive and negative accounts. Interviews were audiotaped, pseudonymized, and transcribed verbatim by trained student research assistants. Transcription followed the rules of Dresing and Pehl, with all transcripts being checked for correctness by CC (23).

### 2.4 Study variables

In addition to demographic data such as age, gender, marital status, living situation, employment status, and education, patients were asked about their disease duration. Cardiac risk factors including smoking, obesity, hypertension, hyperlipidemia, diabetes, and positive family history as well as the presence of any further cardiac or somatic comorbidity were assessed. The severity of cardiac symptoms was measured through the New York Heart Association (NYHA) classification (24) and the Canadian Cardiovascular Society Classification (CCSC) (25). Both are self-reporting questionnaires: in the NYHA classification, patients rate the severity of their dyspnea, whereas the CCSC assesses chest pain. Both classifications categorize patients into four classes, ranging from “no impairment at all” to “impairment even at resting”. With increasing symptom severity, patients are assigned to higher classes. The determination of these classes is based on the criteria outlined in the questionnaires, which consider the reported symptoms, limitations in daily activities, and the impact of the symptoms on the patient's overall well-being.

### 2.5 Data analysis

Data was analyzed according to the qualitative content analysis by Kuckartz (26) and using the software MAXQDA (version 2022). The analysis was conducted by identifying themes at the semantic (explicit) rather than latent (interpretative) level, as we were interested in evaluating the expectation-focused intervention from the patient's perspective. Given the exploratory nature of the research interest, we predominantly used an inductive approach, after initially formulating deductive themes derived from the interview guide and intervention. The analysis process involved multiple stages and collaborative efforts among the research team. The first author (CC) and co-author (SKU) independently conducted an initial data screening. During this phase, we focused on the formulated deductive themes. Gradually, both coders (CC and SKU) began identifying new themes and interesting features not anticipated in the deductive phase. These new themes emerged through an inductive coding process. Subsequently, CC, SKU, and SKO (as the last author) engaged in discussions to create a preliminary codebook. This codebook served as a guide for the subsequent coding process. CC and SKU then systematically coded the entire dataset multiple times, referring to the preliminary codebook. During this process, they engaged in ongoing discussions to resolve any ambiguities or discrepancies in the interpretation of the data. In the final stage of coding, the research team merged or redefined subthemes as needed to ensure the coding accurately reflected the nuances and variations present in the data. This iterative process allowed for a more nuanced and differentiated coding of the material.

## 3 Results

### 3.1 Sample description

Within the sample (*N* = 18), age ranged from 19 to 71 years with a mean of 51.3 years (SD = 15.5). We included *n* = 11 patients of the intervention group who had received the expectation-focused intervention and *n* = 7 patients of the control group who had not received the intervention. Within the sample, 61% of patients (*n* = 11) experienced a long disease duration (more than three years), whereas 39% of patients (*n* = 7) described a short disease duration (less than three years). Of the 18 patients, *n* = 5 patients experienced complications from the heart valve surgery including sternal rewiring, retroperitoneal hematoma, pseudoaneurysm, increased levels of inflammatory markers, single postoperative tachycardic atrial fibrillation, and rethoracotomy. Regarding the type of surgery, *n* = 8 underwent reconstruction, *n* = 9 received a bioprosthetic valve replacement, and *n* = 1 received a mechanical valve replacement. Regarding gender, there was an overrepresentation with 78% male patients (*n* = 14) compared to 22% female patients (*n* = 4). Further relevant characteristics are shown in Table 1.

**Table 1:** Description of the study sample (*N* = 18).

Characteristics (*N* = 18)	
Sociodemographics, *n* (%)	
Age, mean (SD), years	51.3 (15.5)
Gender, female	4 (22.2)
Group affiliation, intervention group	11 (61.1)
Living alone	4 (22.2)
Married	9 (50.0)
≥10 years of formal education	17 (94.4)
Employed	12 (66.7)
Clinical characteristics, *n* (%)	
≥3 years of disease duration	11 (61.1)
No cardiac comorbidities, mean (SD)	17 (94.4)
No somatic comorbidities, mean (SD)	12 (66.7)
Cardiac risk factors, *n* (%)	
Hypertension	6 (33.3)
Diabetes	1 (5.6)
Hyperlipidemia	11 (61.1)
Smoking	1 (5.6)
Obesity	3 (16.7)
Positive family history	14 (77.8)
Angina pectoris, *n* (%)	
CCS Class 1	4 (22.2)
CCS Class 2	2 (11.1)
CCS Class 3	5 (27.8)
CCS Class 4	7 (38.9)
Dyspnea, *n* (%)	
NYHA Class 1	11 (61.1)
NYHA Class 2	5 (27.8)
NYHA Class 3	2 (11.1)
NYHA Class 4	0 (0.0)

CCS, Canadian Cardiovascular Society Classification; NYHA, New York Heart Association Classification.

### 3.2 Patients' evaluation of an expectation-focused intervention in the context of heart valve surgery

**Figure 1:**
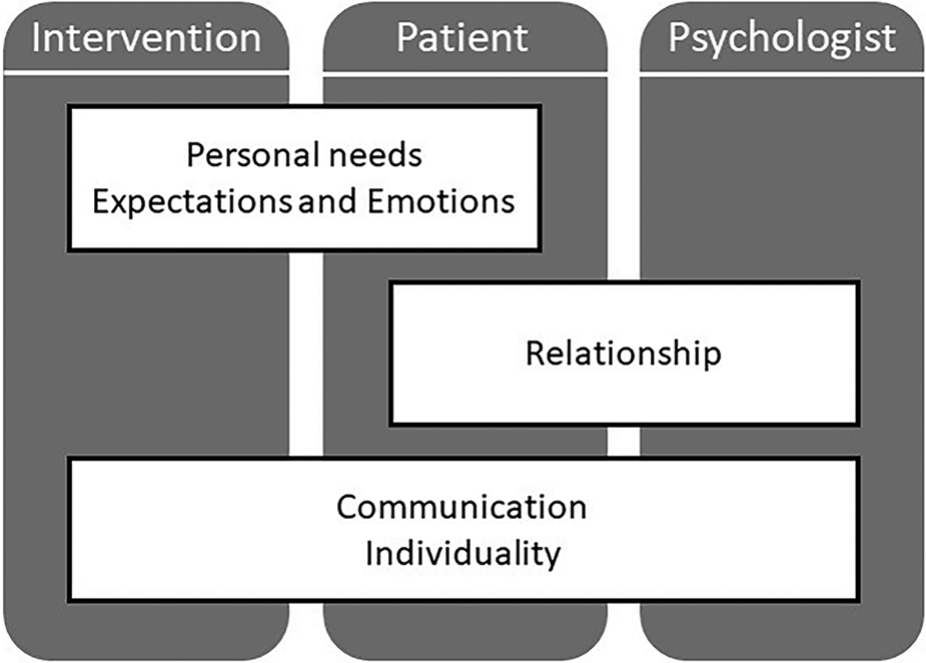
Identified themes from patients’ evaluation of an expectation-focused intervention in the context of heart valve surgery.

The main aim of this qualitative study was to gain insights into how patients undergoing heart valve surgery evaluate an expectation-focused intervention. During the analysis, it became apparent that patients evaluated not only the intervention itself but also assigned importance to the roles of both the patient and the psychologist conducting the intervention. We identified five main themes in the patients' evaluation of the expectation-focused intervention: personal needs, expectations and emotions, relationship, communication, and individuality. As seen in Figure 1, the patient who played a pivotal role in evaluating the expectation-focused intervention was involved in all identified themes. Moreover, certain themes, such as personal needs or expectations and emotions, influenced the content and design aspects of the intervention significantly. Conversely, the psychologist conducting the intervention notably influences the relationship theme. The evaluation of communication and individuality could be observed as being influenced collectively by the patient, the psychologist, and the intervention.

#### 3.2.1 Personal needs

One of the themes most discussed by patients undergoing heart valve surgery was the personal need for an expectation-focused intervention. Patients expressed a range of views on this theme, from feeling no personal need for such an intervention to recommending its implementation for every surgery:

*‘Perhaps it could be different with a more difficult operation or if I'm not doing well, but now in the course of this operation, I didn't really feel the need to get help through personal conversation.’*

*‘Yes, I think that this should happen for every operation. Because every operation is worrying for the patient and their anxiety levels may vary. Therefore, I think it*’*s always appropriate to have such a conversation.’*

Patients' views on the need for an expectation-focused intervention varied, based on how well they were coping with the surgery themselves. For some patients, the decision depended on their individual experiences and symptoms:

*‘When I’m not well, it depends on why I'm feeling that way. If it's due to nausea or pain, I wouldn't necessarily want to talk to a psychologist. If anxiety would overwhelm me, then it would be more likely. It depends on what the symptoms are.'*

The evaluation concerning the time frame of the expectation-focused intervention was one further aspect of personal needs. One patient experienced the session after the surgery too early and recommended more time to focus on themselves. The majority supported the given time frame, including the length and frequency of sessions:

*‘Well-arranged at excellent intervals. Both, before the operation, you were there on the day before and then again afterwards, and again after Christmas. I must say that I found the intervals perfect. Because the phases I was in were very different. My perspective regarding the occurrence changed after the operation.’*

#### 3.2.2 Expectations and emotions

The theme of expectations and emotions primarily focused on content-related aspects of the intervention. In accordance with the aim of the expectation-focused intervention, patients described the preparation for the surgery as helpful. Building up strategies such as how to deal with side effects following the surgery increased the experienced personal control:

*‘I remember our first conversation really well. You advised me to prepare for possible pain and shortness of breath during my treatment but assured me that these symptoms can be managed. Your words helped me understand what to expect and made me feel better prepared for it.’*

Although the patients experienced increased personal control, it seemed difficult for them to build up realistic expectations. Some patients described a discrepancy between their expectations and the perceived reality after the surgery, which was not communicated clearly enough in the intervention.

During the pre- and postoperative phases, patients experienced different emotions such as overburdening, helplessness, and anxiety but also confidence and hope. In addition to preparing for potential somatic side effects, patients found it beneficial to be mentally prepared for the emotional aspects before and after surgery. One example is the relief experienced by the space given for emotions:

*‘It was positive because for me, when talking about critical points or difficult things and then for example an emotion comes through like crying it feels like a release. So it*’*s definitely been positive.’*

#### 3.2.3 Communication

Patients regarded transparent communication as a positive factor and emphasized the importance of language which is not perceived as banalizing throughout the treatment. Concerning the feasibility, the content of the expectation-focused intervention was assessed to be clear and comprehensible. While the content was straightforward to follow, patients encountered uncertainty regarding the purpose of the intervention, leading them to question what personal benefits they could expect:

*‘I'm not quite sure about the objective [of the expectation-focused intervention], so I'm not sure of the best way to achieve it.’*

#### 3.2.4 Relationship

Within the theme of relationship, the perception of the relationship between the psychologist and the patient was evaluated. Patients described a trustful relationship with the psychologist as supportive, viewing them as a contact person with whom they can openly discuss difficulties and worries. Patients deemed a trusting relationship crucial for discussing sensitive and challenging topics without fear of judgment. However, they evaluated the process of establishing this trustful bond with the psychologist as challenging:

*‘[Building up trust towards the psychologist] is not something that can be accomplished in ten or fifteen minutes. You first have to build up a basis of trust in order to show your inner self in a conversation or to show your direct feelings or what is bothering you before the operation. I don't think you can build up this trust in such a short time.’*

Patients described inconsistent evaluations regarding the inclusion of a psychologist into the pre- and postoperative treatment of heart valve surgery. Some patients felt that it carried a sense of stigmatization, while others evaluated the psychologist as an integral part of an interprofessional team, contributing to a holistic treatment approach:

*‘I thought, well, why do I need a psychologist now? […] That was a bit surprising for me.’*

*‘This process of talking to a psychologist is a matter of course for me in the process of good health treatment and even if there is no need, it is still good to have checked it as maybe something is hidden where there is still a need. That*’*s why I approached the conversation with a positive attitude.’*

#### 3.2.5 Individuality

The theme of individuality encompassed patients' evaluation of feeling individually addressed and understood within the intervention. Patients evaluated the individuality of the expectation-focused intervention differently. Some patients felt it catered to their individual needs, while others did not experience this personalization:

*‘No, I didn't really feel personally addressed.’*

*‘I felt like I was receiving totally personalized care. [The psychologist] also knew exactly what my weak points were, which worried me initially. And later they asked if I felt better [concerning the weak points]. And I found that very individual.’*

#### 3.2.6 Additional findings

During the interviews, we encountered results that piqued our interest but were not directly related to the research question. These findings are discussed in the following section, which presents additional findings.

One further aspect of the expectation-focused intervention was to involve close relatives in the process. They could join during the preoperative session, which included the management of expectations concerning the surgery and recovery as well as building up strategies to handle unpleasant symptoms. The participation of close relatives was experienced as supportive by the patients, especially in the sense of having somebody to exchange experiences and emotions with. Additionally, some patients suggested offering sessions with a psychologist to relatives of patients undergoing heart valve surgery.

The expectation-focused intervention was embedded into an interprofessional treatment approach. The interprofessional exchanges, especially during ward rounds where experts from various professions participated, were evaluated as a particularly positive aspect when it comes to a holistic treatment approach.

Within the sample, we identified patients who experienced complications related to heart valve surgery. Their evaluation of the expectation-focused intervention was similar with those of patients without complications.

In addition to the evaluation through patients who had received the expectation-focused intervention, we conducted interviews with patients of the control group. The main challenge was that these patients had not received an expectation-focused intervention and therefore could not evaluate the intervention. To overcome this challenge, we adjusted the interview guide. Instead of evaluating the actual intervention, the control group patients were asked to evaluate the contents and aims which would typically be addressed in the expectation-focused intervention. We were interested in the opinion of patients of the control group as they had not been biased by receiving the expectation-focused intervention beforehand but at the same time were able to assess the experience of a heart valve surgery itself. Concerning the themes of expectations and emotions, relationship, communication, and individuality, the evaluation was congruent with the evaluation described by patients of the intervention group. In respect of personal needs, patients of the control group also varied between no personal need and the view of implementing an expectation-focused intervention in routine care. Without experiencing the interventions, some patients evaluated a higher personal need after the surgery than they would have expected in a preoperative stage.

#### 3.2.7 Overall evaluation of the expectation-focused intervention

All patients (*N* = 18) were asked whether they (a) would themselves participate (again) and (b) would recommend others to participate in the expectation-focused intervention if they were undergoing heart valve surgery. Of the intervention group, 100% of the patients (*n* = 11) agreed they would participate again and would also recommend it to others. Of the control group, 86% of the patients (*n* = 6) stated they would have participated if they had been given the possibility. All patients of the control group (*n* = 7) reported they would recommend the expectation-focused intervention to others undergoing heart valve surgery.

Furthermore, patients were asked about any adverse effects they may have experienced due to the expectation-focused intervention. All patients responded negatively, indicating an absence of any adverse effects. Overall, the patients evaluated the expectation-focused intervention as helpful:

*‘I found the sessions [with the psychologist] really useful because they changed my approach to the whole process of the operation. I also knew that I had support when I wasn't doing well. That*’*s why I wouldn't do without it under any circumstances.’*

### 3.3 Optimization of the expectation-focused intervention

A key consideration in enhancing the expectation-focused intervention for patients undergoing heart valve surgery revolved around addressing personal needs. The aim would be to find strategies to provide support for those who benefit from optimizing expectations while streamlining the intervention for those who do not experience the same level of benefit. According to the patients interviewed, a higher need for support would be relevant for patients with anxiety or negative attitudes towards life, patients of higher age, patients with little social support or self-care, or those experiencing acute symptoms. The patients interviewed assessed patients who knew about their surgery far in advance as having less need for an expectation-focused intervention compared to patients with less preparation time or less experience when it comes to surgery in general.

Concerning the time frame of the intervention the patients evaluated it as helpful that the psychologist took the first step in approaching the patient, offering the expectation-focused intervention. At the same time, they suggested adapting the amount and length of appointments individually, based on what the psychologist and the patient mutually judge to be beneficial:

*‘I would definitely offer this to every patient before and after the operation at certain intervals. And also approach the patient directly, as I think that many patients have an inhibition to seek help and it*’*s easier to accept help when it*’*s offered.’*

Furthermore, the patients evaluated the content of the expectation-focused intervention as well-suited for the context of a heart valve surgery and that none of the addressed topics should have been omitted. To enhance personal control, patients recommend more participation in the surgical process as an integral component of the expectation-focused intervention. This could include options like watching a video of the surgery in advance. Furthermore, the patients addressed the topic of acceptance. They proposed including breathing and relaxation exercises to better handle emotions such as anxiety and helplessness. Apart from discussing the current situation of heart valve surgery, patients expressed a desire to focus on health-related behavior. This could be achieved by including stress management and nutritional information in the intervention. While some patients recommended including discussions about the topic of death within the intervention, others strongly disapproved of this idea. Adding any decision-making processes such as heart valve choice was not mentioned as a topic relevant to the patient in the context of the expectation-focused intervention.

## 4 Discussion and conclusion

### 4.1 Discussion

Overall, the patients experienced the expectation-focused intervention as helpful and none of the patients reported experiencing any adverse effects from it. The results indicate that not only the intervention but also the role of the patient and psychologist play a major part in evaluating the expectation-focused intervention. One of the most discussed themes by the patients is the assessment of personal needs and how these needs vary among patients. As positive aspects, the preparation concerning the surgery and recovery including how to handle unpleasant symptoms as well as the space for emotions are mentioned. It is evaluated positively that the expectation-focused intervention aligned with topics that were individually relevant to the patient. Concerning communication patients assume they would benefit from a clearer understanding of the advantages they can gain from the intervention. Establishing a trustful relationship within the limited time frame before the surgery also appears to be a challenging task.

According to Holmes and colleagues, it is important that patients' discrepancies between pre- and postoperative expectations are as small as possible (10). The patients in this study describe the development of realistic expectations as challenging as the surgery and its consequences could take different courses and individual perceptions seem relevant. Within the expectation-focused intervention, the patients evaluated the preparation and development of strategies to cope with difficulties before and after the surgery as helpful and associated with increased personal control. Kube and colleagues shift the focus toward postoperative expectations when it comes to improving the clinical outcome after surgery (27). Further development of the expectation-focused intervention could focus on postoperative expectations. Furthermore, patients could experience higher personal control by receiving additional insights into the surgery process. Klein and colleagues interviewed patients undergoing radical cystectomy and urinary diversion concerning their perioperative experiences (28). These patients expressed a desire for the implementation of a buddy system. Such a buddy system where patients in the perioperative phase are paired with patients who have undergone successful heart valve surgery for an experience exchange could be considered as a helpful additional element to the expectation-focused intervention.

Patients describe establishing a trustful relationship within the limited time frame before the surgery as a challenge. We deduct that stigmatization associated with sessions with a psychologist is a contributing factor that complicates building up trust and admitting personal needs. Some patients additionally reflected that the purpose of the intervention and their possible personal benefit were not easy to identify. During the initial session of the expectation-focused intervention, the psychologist introduces and discusses the concept and purpose. To align the purpose with the individual needs of the patient, it could be beneficial to provide a more detailed and stronger differentiated explanation about the topics encompassed within the intervention. Furthermore, the psychologist's role could be introduced in a manner that avoids the use of language or terminology that might contribute to stigmatization.

When it comes to the experienced individuality of the expectation-focused intervention, the majority of patients perceive it as addressing their individual needs. Table 1 illustrates variations among the patients undergoing heart valve surgery, including variances in disease duration, comorbidities, cardiac risk factors, as well as other clinical characteristics like angina pectoris and dyspnea. However, it remains unclear which patients derive the greatest benefit from the intervention. Future research should place a greater emphasis on understanding the specific needs of various patient subgroups while actively involving patients in the process of the intervention.

The patients in this study suggested incorporating additional elements into the expectation-focused intervention, such as including breathing exercises and learning new strategies to cope with stress. To face the challenge of maintaining an efficient time schedule, it could be valuable to organize the expectation-focused intervention as a modular concept. The choice of modules on which to focus could be determined by the patient's preferences and what appears relevant from the psychologist's perspective, possibly, for example through the use of screening tools. Auer and colleagues provided initial insights into tailored approaches within expectation-focused interventions (12). Their study revealed that patients seeking to alleviate depressive symptoms should focus on different aspects of expectations, whilst focusing on expected consequences is particularly beneficial in reducing anxiety (12). In future studies, to obtain a more comprehensive perspective, it is essential to include a diverse range of patients with varying sociodemographic, disease-related, and psychosocial variables. Quantitative measures related to the identified themes could be used to test associations between these variables and the evaluation of the expectation-focused intervention. A more nuanced understanding of which patient subgroups profit from specific intervention focuses and frequencies could also lead to a more efficient allocation of resources within the healthcare system.

### 4.2 Limitations

The results of this study should be interpreted in light of the following limitations. Firstly, the interview subsample was self-selected. The patients of the intervention group were asked during the initial session of the expectation-focused intervention whether they would like to participate in the interview to evaluate the intervention, whereas patients of the control group were approached after the surgery. All patients provided informed consent and retained the option to withdraw from the study at any stage. Although no patients chose to withdraw, it is possible that those who participated may have had a higher level of motivation to invest effort in evaluating and improving expectation-focused interventions. Furthermore, the sample included more male patients (78%) in comparison to female patients (22%). An overrepresentation of male patients is a recognized characteristic in the context of heart valve surgery, which is why we consider the collected data sufficient in terms of achieving a balanced sample, based on pre-defined criteria (29).

Secondly, the sample was recruited from an RCT which included repeated assessments for all patients and increased interprofessional treatment for the intervention group. The assessments comprised surveys including questions about mental health which might have induced heightened self-reflection when compared to patients not participating in the study. By including patients of the control group in the qualitative study, we obtained insights into patients who did not receive enhanced interprofessional treatment but nevertheless underwent heart valve surgery. These patients did not receive the expectation-focused intervention which helps mitigate bias but also poses a challenge in envisioning the potential impact of such an intervention. Moreover, the patients may have faced challenges in distinguishing between the various interprofessional aspects of the treatment. To assist patients in identifying the components associated with the expectation-focused intervention, we revised the specific elements linked to this intervention at the beginning of the interview.

Thirdly, due to the protection of data, the interviews were solely conducted with patients from the University Medical Center Hamburg-Eppendorf. This limits the choice of psychologists performing the expectation-focused interventions with the patients. Both the performance of the intervention and the evaluation process were executed by the Department of Psychosomatic Medicine and Psychotherapy at the University Medical Center Hamburg-Eppendorf. By involving different members of the department in the process of conducting the intervention and interviews, transcribing, coding, and analyzing the interviews, we aimed to reach the highest possible validity within our resources.

## 5 Conclusion

Overall, our analysis generated first insights into patients' views on expectation-focused intervention in the context of heart valve surgery. This qualitative study shows an overall endorsement of the intervention and appears to address holistic care demands. Establishing a trustful relationship and perceived stigmatization of treatment by a psychologist pose the main challenges. Stigmatization could be reduced by framing the role of the psychologist differently and implementing an expectation-focused intervention as part of routine care. The initiation of initial contact could be beneficial in reducing stigmatization and lowering the barrier to seeking help. To address individual needs in the best possible way, the introduction of a modular concept, where patients and psychologists in collaboration determine the most suitable focus and frequency of the intervention, could be considered. Certain topics, such as preparing for unpleasant symptoms and providing space for emotions, should be integral components of every intervention. Innovative enhancements, such as providing sessions for relatives, and expanding the range of topics to include breathing exercises and stress-coping strategies, could be considered as additions. Introducing a buddy system, pairing patients in the preoperative phase with those who have successfully undergone heart valve surgery to enhance experience exchange, could also be of great value. Future research should concentrate on developing the expectation-focused intervention within a modular concept and investigate the influence of sociodemographic, disease-related, and psychosocial variables in identifying specific personal needs among patient subgroups.

## Data Availability

The raw data supporting the conclusions of this article will be made available by the authors, upon reasonable request.
